# Antimicrobial Effects of Three Plant-Derived Phenolic Compounds and Their Potential Role in Strawberry Preservation

**DOI:** 10.3390/foods14234142

**Published:** 2025-12-03

**Authors:** Ziwei Liang, Shengshuai Li, Lanxi Zhang, Fengqin Wu, Shuyan Pu, Xinyue Liu, Yu Rao

**Affiliations:** Food Microbiology Key Laboratory of Sichuan Province, Chengdu 610039, China

**Keywords:** plant-derived phenolic compounds, resveratrol, epigallocatechin gallate, tea polyphenols, microbial spoilage, strawberry preservation

## Abstract

Microbial spoilage of nutrient-rich strawberries leads to considerable food waste and economic losses. Plant-derived phenolic compounds, including resveratrol (RES), epigallocatechin gallate (EGCG), and tea polyphenols (TP), have gained attention for their multi-target antimicrobial efficacy and potential applications in fruit preservation. This study evaluated the individual and combined effects of these three compounds on strawberries infected with *Escherichia coli* (*E. coli*) and *Botrytis cinerea* (*B. cinerea*). The minimum inhibitory concentration (MIC) values for RES (analytical grade, ≥99% purity) and EGCG (analytical grade, ≥98% purity) against *E. coli* were 1.56 g/L and 25 g/L, with an additive effect against *E. coli* growth (FICI = 0.625). 5 g/L TP (analytical grade, ≥98% purity) completely inhibited the mycelial growth of *B. cinerea*. The in vivo application of RES and EGCG significantly reduced spoilage and improved texture, color, weight retention, and flavor quality in strawberries infected by *E. coli* individually or in combination. Similarly, the combined use of TP and chitosan saved the quality of strawberries infected by *B. cinerea* compared to single treatments. This study provided new effective and eco-friendly strategies for the preservation of strawberries.

## 1. Introduction

Strawberries represent a globally important berry crop with considerable economic value, and their market scale continues to expand—global sales of fresh strawberries are projected to hit $18 billion in 2024 and further rise to $23 billion by 2030 [1]. As one of the fruits with the highest yield per unit area, strawberries have even been dubbed the “golden industry” in the fruit economy, underscoring their role in driving agricultural economic growth [2]. However, their high nutrient content and fragile epidermal structure render them extremely vulnerable to microbial spoilage, which leads to severe postharvest losses: typically 25–40% during circulation, and up to 50% in regions with underdeveloped cold chain systems [3]. These losses translate to an estimated $8 billion in annual economic damage, while also worsening issues like price fluctuations and seasonal supply shortages in the market [4]. Against this backdrop, developing innovative preservation technologies is essential to safeguarding the profitability of the strawberry industry. For instance, optimized management of key links—including harvesting, pre-cooling, packaging, storage, and transportation—can extend the commercial shelf life of strawberries from the original 3–5 days to 7–15 days [5]. Notably, research focusing on microbial spoilage is of particular significance: it not only directly clarifies the mechanisms underlying the accelerated decline of strawberry quality but also lays a critical foundation for enhancing preservation efficiency and stabilizing the year-round market supply of strawberries.

*Escherichia coli* (*E. coli*), a prominent foodborne pathogen within the *Proteobacteria* phylum linked to strawberry spoilage [6] exerts a significant threat to strawberry quality by infiltrating the fruit’s tissues through surface wounds or stomata. A key driver of its spoilage capacity is Curlin subunit gene D (CsgD): as a transcriptional activator in *E. coli.* CsgD regulates the synthesis of curli fimbriae and cellulose, which in turn enhance the bacterium’s adhesion to strawberry surfaces and promote biofilm formation [7]. This biofilm not only stabilizes *E. coli*’s colonization but also shields it from environmental stress, ultimately accelerating fruit decay and causing substantial deterioration in strawberry quality [8]. *Botrytis cinerea* (*B. cinerea*), the most economically impactful fungal pathogen among strawberry-spoiling fungi [9], drives spoilage through a targeted enzymatic mechanism. *B. cinerea* secretes a suite of cell wall-degrading enzymes, such as pectinases and cellulases, that specifically hydrolyze the structural polysaccharides in strawberry tissues. This enzymatic breakdown directly manifests as the characteristic gray mold lesions, a hallmark symptom of fungal-induced decay that severely impairs strawberry quality [10].

Li et al. reported that the physical environmental regulation of low oxygen combined with high CO_2_ suppresses strawberries’ respiratory intensity, reduces metabolic consumption, maintains relatively high fruit pH and firmness, and delays structural deterioration [11]. It was proved that 250 Hz acoustic wave treatments for 10 days could inhibit the growth of spoilage fungi on strawberries, effectively extending the fruit’s shelf life [12]. Though environmentally friendly, these physical strategies have drawbacks: high operational complexity, equipment dependence, and limited antimicrobial effects [13,14]. Fungicide boscalid and difenoconazole reduced the contents of soluble sugars and nutrients, increased organic acid levels, and caused oxidative damage in mature strawberries, ultimately impairing their flavor quality [15]. Carbendazim inhibits fungal cell division by targeting fungal β-tubulin to exert its antifungal effect, but currently, the fungi causing postharvest rot in strawberries have developed varying degrees of resistance to it [16]. However, chemical agents for fruit preservation not only pose adverse impacts to soil and water, disrupt ecological balance, and risk biomagnification in food chains, but also drive the development of resistance in target fungi—undermining their long-term effectiveness [17,18]. Therefore, the application of natural products in strawberry preservation represents a highly promising and underdeveloped strategy.

Resveratrol (RES) is a phenolic compound mainly derived from plants such as grape skins, tiger cane, and berries, containing the characteristic unit of two phenolic hydroxyl groups directly attached to a stilbene backbone [19,20]. RES induces reactive oxygen species accumulation in *Salmonella typhimurium*, resulting in increased membrane permeability, DNA damage, and ultimately leading to cell death [21]. Tea polyphenols (TP) are a phenolic mixture mainly from tea, including key catechins, flavonoids and phenolic acids, possessing antioxidant, antibacterial and fruit ripening-regulating activities [22]. Epigallocatechin gallate (EGCG), as the most active component of TP originated from green tea, has a polyphenolic structure with 8 aromatic ring-attached hydroxyl groups and a galloyl moiety [23,24]. TP effectively controls gray mold-induced necrotic rot in nectarines, with in vitro assays confirming dose-dependent mycelial growth inhibition and in vivo applications demonstrating significant reduction in both disease incidence and lesion diameter [25]. Wang et al. reported that EGCG combined with hyaluronic acid exhibits superior preservation effects on Torreya grandis kernels than those used alone [26]. Hu et al. found that trans-resveratrol combined with a chitosan matrix outperforms it standalone in strawberry preservation, effectively reducing dehydration, inhibiting microbes, preserving quality and extending shelf life at 22 °C and 4 °C [27].

Owing to the limited preservation efficacy of single compounds, combinations of different phenolic compounds have been applied to evaluate their effects on fruit preservation. The fractional inhibitory concentration index (FICI) is used to assess the synergistic effects between various agents, with its values interpreted as follows: FICI ≤ 0.5 indicates synergistic effect indicates combined inhibition stronger than the sum of individual effects; 0.5 < FICI ≤ 1.0 denotes additive effect implies combined inhibition equals the sum of individual contributions; 1.0 < FICI ≤ 4.0 signifies no interaction; 4.0 < FICI represents antagonistic effect suggests combined inhibition weaker than individual effects [28].

This study aims to explore the antimicrobial effects of RES, EGCG, and TP in vitro, and their preservation effects on strawberry in vivo. We determined the antimicrobial activities of RES, EGCG, and TP against *E. coli* and *B. cinerea*. Then, we evaluated how these compounds, applied singly or in combination, influenced the hardness, weight loss, color retention, sensory qualities, and nutritional composition of strawberries stored in natural environment of infected by *E. coli* or *B. cinerea*. This study will provide data support for further exploring their potential in targeted scenarios.

## 2. Materials and Methods

### 2.1. Strawberries, Strains, Mediums and Chemicals

The strawberries used were of the “Hongyan” cultivar, harvested at commercial maturity from a local organic farm in Chengdu, Sichuan Province, and transported to the laboratory within 4 h post-harvest.

The microbial strains employed in this study, specifically *E*. *coli* (DH5α), *B*. *cinerea*, *Colletotrichum gloeosporioides* (*C. gloeosporioides*), and *Aspergillus niger* (*A. niger*) were sourced from the laboratory’s internal culture collection and preserved at −80 °C in 15% (*v*/*v*) glycerol (Chengdu, China). Key bioactive compounds, including resveratrol (RES, analytical grade, ≥99% purity), epigallocatechin gallate (EGCG, analytical grade, ≥98% purity), and tea polyphenols (TP, analytical grade, ≥98% polyphenol content), were procured from Shanghai Macklin Biochemical Co., Ltd. (Shanghai, China). Medium molecular weight chitosan, with a deacetylation degree of 85 ~ 90%, was obtained from Shanghai Yuanye Bio-Technology Co., Ltd. (Shanghai, China). The following culture media and supporting materials were obtained from their respective suppliers: Mueller–Hinton broth (MH) from Qingdao Hope Bio-Technology Co., Ltd. (Qingdao, China); Potato Dextrose Agar (PDA) and Potato Dextrose Broth (PDB) from Beijing AoBoxing Bio-Technology Co., Ltd. (Beijing, China) and bacteriological agar from Beijing Solarbio Science & Technology Co., Ltd. (Beijing, China). Critical reagents for buffer preparation, such as sodium hydroxide (analytical grade, Tianjin Dalu Chemical Reagent Factory) (Tianjin, China), Tween-80 (pharmaceutical grade, Tianjin Komiou Chemical Reagent Co., Ltd.) (Tianjin, China), and phosphate-buffered saline (PBS, pH 7.4 ± 0.1, Beijing Lanjieke Technology Co., Ltd. (Beijing, China)), were purchased from the indicated commercial sources. All working solutions were prepared using ultrapure water and sterilized by filtration through 0.22 μm membranes prior to microbiological assays.

### 2.2. Minimum Inhibitory Concentration (MIC)

The MIC of RES and EGCG against *E. coli* was determined in triplicate by broth microdilution in accordance with Clinical and Laboratory Standards Institute guidelines (CLSI M07-ED12) [29]. In brief, serial two-fold dilutions of RES and EGCG were prepared in a 96-well plate with sterile Mueller–Hinton broth. Then 100 μL of the 10^6^ CFU/mL *E. coli* inoculum was added to each well (final concentration at 5 × 10^5^ CFU/mL), followed by incubation at 37 °C for 20 h. The MIC was the lowest concentration of RES/EGCG that inhibited visible bacterial growth.

The MIC value of TP against *B. cinerea*, *C. gloeosporioides* and *A. niger* was evaluated by the mycelial growth rate method [30]. A 20 g/L stock TP solution was added to PDA medium to prepare final concentrations of 0.16–10 g/L. Aseptically, 5 mm-diameter mycelial plugs were inoculated onto the center of TP-amended PDA plates, with the mycelial side facing down. All treatments were performed in triplicate, using TP-free PDA plates as controls. Plates were incubated at 25 °C in the dark for 3 days. Fungal growth was evaluated by measuring the mycelial diameter of *B. cinerea* at two perpendicular directions using a ruler. The average of these two measurements was used to calculate the inhibition rate of mycelial growth according to the following formula:

*IR* (%) = (*C* − *T*)/*C* × 100% (1)

where *IR* stands for mycelial inhibition rate, *C* mean radial mycelial growth of control group, *T* mean radial mycelial growth of treated samples. The lowest concentration of a drug that completely inhibits fungal growth is defined as the MIC.

### 2.3. Fractional Inhibitory Concentration Index (FICI)

The combined antibacterial effect of RES and EGCG against *E. coli* was evaluated using an adapted checkerboard microdilution method, as previously described [31]. In this system, the two compounds were arranged along the orthogonal dimensions of a 96-well plate: EGCG was diluted serially along the rows, and the other along the columns, enabling systematic testing of all concentration combinations. The initial concentrations of RES and EGCG were consistent with those previously determined in individual MIC assays. Each test plate contained control wells with each agent alone, as well as growth medium controls. *E. coli* cultures in the logarithmic growth phase were diluted and inoculated at 100 μL per well, achieving a final cell density of 5 × 10^5^ CFU/mL. After 24 h of incubation at 37 °C, the FICI value was calculated to quantify the interaction between EGCG and RES according to established formulas:

(2) $$FICI=FICI_{A}+FICI_{B}=\left({C_{A}}^{COMB}/MIC_{A}\right)+\left({C_{B}}^{COMB}/MIC_{B}\right)$$

where *MIC_A_* and *MIC_B_* are defined as the MICs of antimicrobials EGCG and RES acting individually, *C_A_^COMB^* and *C_B_^COMB^* are the concentrations of EGCG and RES when used in combination. FICI ≤ 0.5: synergistic effect indicates combined inhibition stronger than the sum of individual effects; 0.5 < FICI ≤ 1.0: additive effect implies combined inhibition equals the sum of individual contributions; 1.0 < FICI ≤ 4.0: no interaction; 4.0 < FICI: antagonistic effect suggests combined inhibition weaker than individual effects.

### 2.4. Growth Curve of E. coli

The growth inhibitory effect of the EGCG and RES was assessed through bacterial growth curve analysis. Following an adapted protocol derived from [32], four groups were established: Group A (1/2 MIC of RES), Group B (1/2 MIC of EGCG), Group C (1/2 MIC RES + 1/2 MIC EGCG), and Group D (untreated control). Each treatment consisted of 30 mL of MH inoculated with 300 μL of bacterial suspension. The experiment included three replicates per group, totaling 12 culture flasks. All flasks were incubated at 37 °C with shaking at 180 rpm/min. Bacterial growth was monitored over 24 h by measuring the optical density at 600 nm (OD_600_) at 2 h intervals.

### 2.5. Spore Germination of B. cinerea, C. gloeosporioides and A. niger

The spore inhibition activity of TP against three fungal pathogens (*B. cinerea*, *C. gloeosporioides*, and *A. niger*) was evaluated by microbroth dilution assay [33]. Spore suspensions (10^6^~10^7^ CFU/mL) obtained from PDA plate cultures were tested in 96-well plates containing PDB medium supplemented with TP at concentrations from 0.39 to 25 g/L. Each well was inoculated with 1% (*v*/*v*) of spore suspension and incubated statically at 25 °C for 24 h in the dark. Absorbance was measured at 530 nm (OD_530_), with appropriate controls and three replicates included for each treatment.

### 2.6. Molecular Docking

Following the methodology outlined by Morris et al. with minor modifications [34], the three-dimensional chemical structures of RES and EGCG were retrieved from the PubChem database (https://pubchem.ncbi.nlm.nih.gov/ (accessed on 19 July 2025)). Homology modeling of the target proteins was conducted using the Swiss-Model platform (https://swissmodel.expasy.org/ (accessed on 19 July 2025)). Potential target proteins associated with growth, division, and biofilm formation in *E. coli* were selected, including sensor histidine kinase CpxA, the DNA-binding transcriptional dual regulator CsgD, L,D-transpeptidase LdtD, cell division protein FtsZ, ATP synthase F1 complex subunit γ, enoyl-[acyl-carrier-protein] reductase, 3-oxoacyl-[acyl-carrier-protein] reductase FabG, dihydrofolate reductase, and DNA gyrase subunit B, with RES and EGCG serving as ligand molecules. For *B. cinerea*, proteins implicated in the degradation of fruit and vegetable cell walls were targeted, including chitin synthase A, chitin synthase, and cutinase, with EGCG as the ligand. Molecular docking was performed using AutoDock Vina 1.5.7 to evaluate the interactions of resveratrol and EGCG with the proteins’ active sites, the results of which were visualized using PyMOL 3.0.3. Binding affinity (kcal/mol) was used as the evaluation metric, with more negative values indicating greater conformational stability.

### 2.7. Effects of EGCG, RES and TP on Infected Strawberries

#### 2.7.1. Treatment of Strawberries

For both *E. coli* and *B. cinerea* infected models [35], there were 8 treatments groups in total (including controls). Each group contained 12 strawberries (to ensure sufficient samples for continuous measurement over 5 days). For biological replication, 3 representative strawberries were selected from each group’s 12 samples for instrumental measurements (with 4 replicate measurements per strawberry, as detailed in the Methods section), resulting in 96 strawberries in total. And the 3 representative strawberries per group were chosen based on strict uniformity (size, color, and surface scratches) to avoid individual differences affecting results.

The strawberries were washed, and air-dried at room temperature (25 ± 2 °C) under ambient humidity (45–55%) for 30 min, then inoculated with 20 μL of the bacterial suspension or 10 μL of spore suspension at concentration of 10^5^~10^6^ CFU/mL [36].

#### 2.7.2. Hardness

The hardness of strawberries were measured every 24 h from day 0 to day 5 using a texture analyzer in TPA (Texture Profile Analysis) mode [37]. For each sampling time point, three replicate measurements were conducted per strawberry sample, and the final recorded data represented the average value of these triplicates. The specific test parameters were set as follows: Probe P/2; pre-test speed, test speed, and post-test speed all set at 5 mm/s; strain rate of 20%; strain duration of 3.0 s; trigger force of 5.0 g.

#### 2.7.3. Weight Loss

The weight changes in strawberries during the storage under different treatments were determined according to protocol of Shi et al. with minor modifications [38]. Strawberries in each treatment group were weighed individually using an electronic balance with a precision of 0.001 g at the initial storage stage (Day 0) and the end of each preset storage period. The weight loss rate was calculated as follows:

(3) $$\mathrm{WL}\left(\%\right)=\frac{W_{0}-W_{t}}{W_{0}}\times 100\%$$

where WL is weight loss, *W*_0_ is initial weight (g), *W_t_* is weight at time t (g).

#### 2.7.4. Color

The color of strawberry was measured using WS70 Spectrophotometer (Weifu Optoelectronic Technology Co., Ltd., Suzhou, China) according to the CIE Lab system where L* represents lightness, and positive values of a* and b* indicates red and yellow colors [39]. Strawberries from both infected and non-infected groups treated with different compounds were selected. For each strawberry, three random positions on its equatorial region were chosen to measure the daily changes in strawberry color during the 5 days storage period, so as to evaluate the effect of different treatments on strawberry color.

### 2.8. Titratable Acid (TA) and Soluble Solids Content (SSC)

Strawberries were blended with sterile water at a 1:2 (*v*/*v*) ratio and filtered through two layers of gauze. For TA measurement, 2 mL of strawberry juice supernatant was mixed with 30 μL phenolphthalein indicator and titrated with 0.5 mol/L NaOH until a persistent pink color appeared (30 s). TA was calculated as follows [40]:

(4) $$\mathrm{TA}\left(\%\right)=\frac{V\;\times \;c\;\times \;\left(V_{\;1\;}-V_{0\;}\right)\;\times \;f\;}{V_{s}m}\;\times \;100\%$$

where *V* = total volume of sample (mL), *c* = NaOH concentration (0.5 mol/L), *V*_1_ = volume of NaOH consumed in titration (mL), *V*_0_ = volume of NaOH consumed in blank control (mL), *f* = conversion factor (0.067 for malic acid, g/mmol), *V_s_* = volume of sample (mL), *m* = sample mass (g).

For SSC, a refractometer was calibrated with distilled water, and 300 μL of filtered strawberry juice was measured three times, with the average value recorded [41]. Strawberry samples were homogenized and then filtered to remove pulp residues, yielding clear juice. A 300 μL aliquot of this filtered juice was applied to the refractometer’s detection prism, and SSC readings were taken three times per sample. The average value of the three measurements was recorded as the final SSC result for the samples.

### 2.9. Sensory Evaluation

Sensory evaluation was performed by a ten-member semi-trained panel using an adapted method from Tomadoni et al. [42]. Strawberry samples were placed in uniform white plastic plates coded with random numbers to avoid evaluator preference and presented to the panel under consistent natural light at 25 °C. Each evaluator scored independently according to the detailed sensory criteria in Table S1 without discussion, and the final sensory score for each sample was the average value of the 10 evaluators’ scores.

### 2.10. Statistical Analysis

All experiments were conducted in triplicate and the data were expressed as mean ± SD. Prior to statistical analysis, the assumption of variance homogeneity for the data was validated using Levene’s test. Statistical analyses were conducted using SPSS software (Version 26.0). For data with homogeneous variances, differences between groups were determined via one-way analysis of variance (one-way ANOVA), followed by Duncan’s multiple range test. For data with non-homogeneous variances, Welch’s ANOVA was used instead, with Games-Howell test for post hoc comparisons. All graphs were generated using Origin software (Version 2021b).

## 3. Results and Discussion

### 3.1. Resveratrol and Epigallocatechin Gallate Inhibited the Growth of E. coli

**Figure 1:**
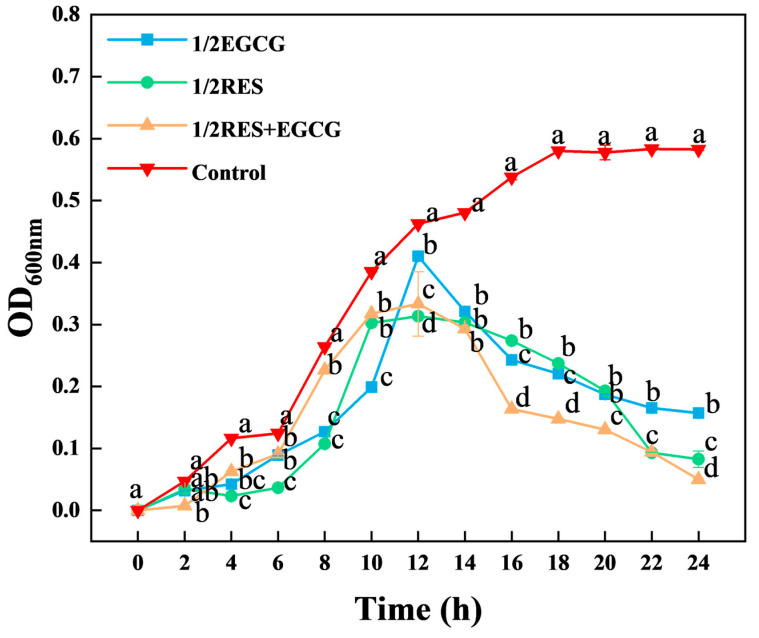
Effects of RES and EGCG on the growth of *E. coli*. Group designations: Control (no drug), 1/2 EGCG (1/2 MIC of EGCG), 1/2 RES (1/2 MIC of RES), and 1/2 RES+EGCG (combination of 1/2 MIC of both RES and EGCG). Data are presented as mean ± SD (*n* = 3). Different lowercase letters above bars denote significant differences (*p* < 0.05). RES: Resveratrol; EGCG: Epigallocatechin Gallate.

In vitro antibacterial tests showed the MICs of resveratrol (RES) and epigallocatechin gallate (EGCG) against *E. coli* were 1.56 g/L and 25 g/L, respectively. Notably, their combination had a FICI of 0.625, indicating an additive antibacterial effect. As shown in Figure 1, *E. coli* growth was sustainably suppressed for 24 h when exposed to the MICs of either compound and the control had significantly higher OD_600_ throughout. Among all treatments, the combination of 1/2 MIC of each compound induced the strongest growth suppression (*p* < 0.05). After 12 h of incubation, single and combined treatments both reduced OD_600_ significantly, shortened the stationary phase, and triggered early entry into the decline phase—while the control maintained stable turbidity.

Consistent with the findings in this study, EGCG has been reported to induce intracellular reactive oxygen species accumulation, which triggers oxidative stress and suppresses the proliferation of *E. coli* [43]. Previous research has shown that RES inhibited the growth of *E.coli* by interfering formation of Z-ring which was a key structure for cell division [44]. The combination of RES and EGCG exerted a more significant antibacterial effect, which is supported by a study showing that the equimolar mixture of resveratrol and kaempferol exhibits synergistic antibacterial activity against *E. coli* [45].

### 3.2. Tea Polyphenols Inhibited the Growth of Spoilage Fungus

**Figure 2:**
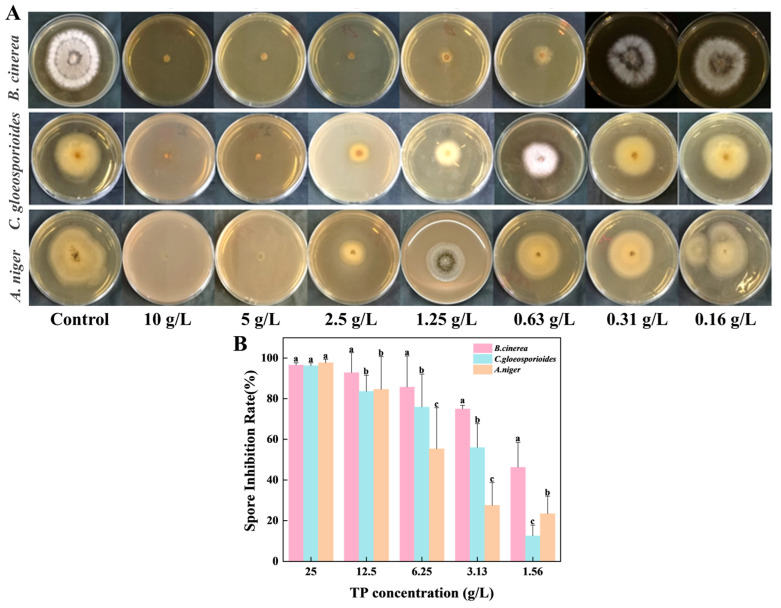
Inhibitory effects of TP on the growth of three spoilage fungi. (**A**) Inhibitory effects of different concentrations of TP on the mycelial growth of *B. cinerea*, *C. gloeosporioides*, and *A. niger*. (**B**) Inhibitory effects on the spore germination of *B. cinerea* (pink), *C. gloeosporioides* (blue), and *A. niger* (orange). Data are presented as mean ± SD (*n* = 3). Different lowercase letters indicate significant differences (*p* < 0.05). TP: Tea Polyphenols.

Tea polyphenols (TP) exhibited dose-dependent suppression of mycelial growth against three postharvest pathogenic fungus: *B. cinerea, C. gloeosporioides*, and *A. niger* (Figure 2A). The MIC of TP was established at 5 g/L for *B. cinerea* and 10 g/L for both *C. gloeosporioides* and *A. niger*. Following 72 h incubation, control groups developed extensive mycelial colonization with diameters of 40 mm, 35 mm, and 42 mm for *B. cinerea, C. gloeosporioides*, and *A. niger*, respectively (Table S2). Notably, TP at 0.63 g/L and 2.5 g/L concentrations inhibited *B. cinerea* growth by 45% and 91.4%, while for *C. gloeosporioides*, concentrations of 2.5 g/L and 5 g/L achieved inhibition rates of 72.6% and 93.8%, respectively, accompanied by morphological abnormalities including hyphal apex swelling and filament fragmentation. Against *A. niger*, TP demonstrated 88.2% mycelial growth inhibition at 5 g/L with complete suppression occurring at 10 g/L. Comparative analysis revealed *B. cinerea* as the most TP-sensitive among the tested fungal species.

As shown in Figure 2B, TP treatment induced concentration-dependent suppression of spore germination across all three fungal pathogens (*p* < 0.05), with *B. cinerea* displaying the greatest susceptibility and *A. niger* the highest resistance. At 1.56 g/L, germination inhibition rates reached 46.3% for *B. cinerea*, 12.5% for *C. gloeosporioides*, and 5.3% for *A. niger*. These values increased to 75%, 56%, and 27.5%, respectively, at 3.13 g/L, demonstrating progressively enhanced efficacy. Complete growth suppression (>90% inhibition) was achieved at 25 g/L for all species, while concentrations exceeding 12.5 g/L already provided over 80% control efficacy, with *B. cinerea* reaching 92.9% inhibition at this threshold.

The mycelial growth of *B. cinerea* could be inhibited by ROS accumulation, which resulted in the damage of cell membrane structure [46,47]. In this study, TP effectively affected the transmission and infection ability by suppressing the conidia germination of *B. cinerea* [46]. To further validate how TP inhibited the mycelial growth and spore germination in *B. cinerea*, further study should be addressed. Among the three fungal species, *B. cinerea* showed the highest sensitivity to TP treatment, evidenced by the lowest MIC value. TP treatment reduced rhizopus rot in nectarines by inhibiting the mycelial growth and spore germination of *Rhizopus stolonifer* [48]. Liu et al. suggested that TP could suppress spore germination and mycelial growth of *B. cinerea* by increasing populations of *Hanseniaspora uvarum* [49]. This highlights the importance of combining TP with biocontrol agents for alleviating gray mold caused by *B. cinerea*.

### 3.3. The Potential Targets of the Phenolic Compounds

**Figure 3:**
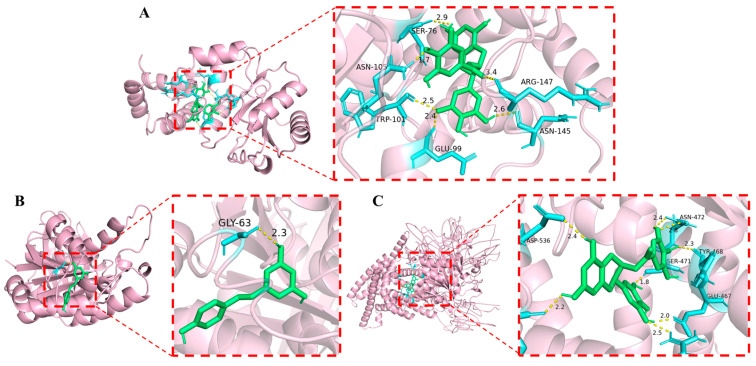
Molecular docking results. (**A**) CsgD, (**B**) FtsZ, (**C**) Chitin synthase A. Green color indicates the phenolic compound; Yellow color denotes hydrogen bonds; Blue color represents the linked amino acids.

Molecular docking analysis was performed to evaluate the binding affinities of EGCG with key regulatory and enzymatic proteins in *E. coli*, revealing substantial binding energies of −6.4 kcal/mol with histidine kinase CpxA, −10.2 kcal/mol with the transcriptional regulator CsgD, −8.1 kcal/mol with L,D-transpeptidase LdtD, −9.7 kcal/mol with enoyl-acyl carrier protein reductase FabI, and −8.5 kcal/mol with biotin carboxylase FabG, with CsgD demonstrating the strongest interaction (Figure 3A). As a master regulator of curli fimbriae and cellulose production, CsgD binding by EGCG is predicted to impair bacterial colonization and biofilm development, consistent with previous findings that EGCG disrupts amyloid fiber assembly and activates the σE-mediated envelope stress response [50]. This response downregulates CsgD expression via the σE-dependent sRNA RybB, which targets CsgD mRNA for degradation, ultimately suppressing biofilm formation and bacterial growth [51].

In the case of RES, the strongest binding was observed with the cell division protein FtsZ (−6.2 kcal/mol) (Figure 3B), followed closely by ATP synthase F1 complex subunit γ (−6.1 kcal/mol) and dihydrofolate reductase (−6.0 kcal/mol). Previous studies indicate that RES inhibits bacterial proliferation by suppressing FtsZ expression and preventing Z-ring formation [44]. As a key component of the bacterial divisome, FtsZ coordinates new septal peptidoglycan synthesis—essential for cell wall constriction and cytokinesis [52]. Thus, the strong binding affinity between RES and FtsZ supports its potential role as an inhibitor of cell division.

Given that EGCG is the primary active component of TP, the potential binding affinity between EGCG towards proteins in *B. cinerea*, including chitin synthases A and chitin synthases, as well as cutinase CutA was analyzed by molecular docking (Figure 3C). The binding energies were −9.9 kcal/mol for chitin synthase A, −8.6 kcal/mol for chitin synthase, and −6.9 kcal/mol for CutA. The strongest binding was observed with chitin synthase A, which is critical for apical hyphal growth and pathogenicity in *B. cinerea*, playing a vital role in cell wall formation [53]. Taken together, the potential antimicrobial target of EGCG against *E. coli* and *B. cinerea* are CsgD and chitin synthase A, while that of RES against *B. cinerea* is FtsZ.

### 3.4. Epigallocatechin Gallate, Resveratrol, and Tea Polyphenols Maintained the Hardness and Weight of Strawberry

**Figure 4:**
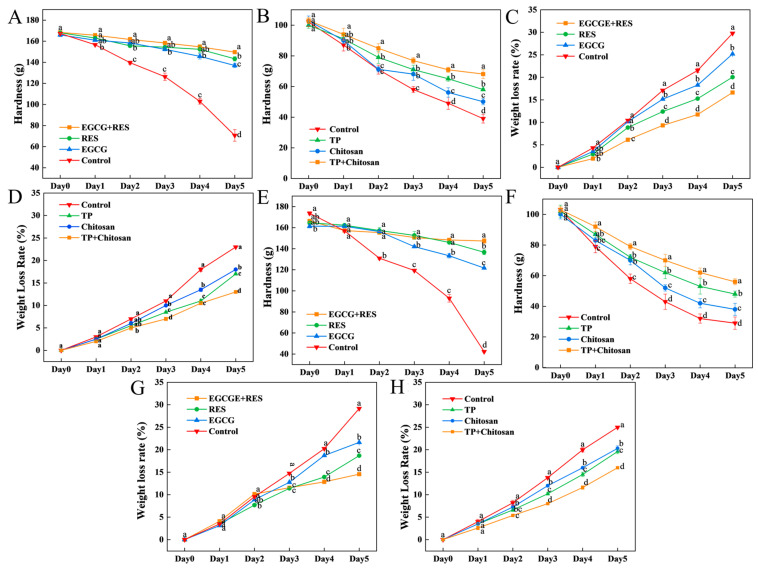
Effects of RES, EGCG, and TP on the hardness and weight loss rate of strawberries. Hardness of strawberries treated with RES/EGCG (**A**) and TP/chitosan (**B**) in uninoculated groups. Weight loss of strawberries treated with RES/EGCG (**C**) and TP/chitosan (**D**) in uninoculated groups. Hardness of strawberries inoculated with *E. coli* (**E**) or *B. cinerea* (**F**) and treated with the respective compounds. Weight loss of strawberries inoculated with *E. coli* (**G**) or *B. cinerea* (**H**) and treated with the respective compounds. Error bars represent SD (*n* = 3). Different lowercase letters indicate significant differences (*p* < 0.05) between treatments on the same day. RES: Resveratrol; EGCG: Epigallocatechin Gallate; TP: Tea Polyphenols.

The textural hardness of strawberries during storage, as quantified in Figure 4A,B,E,F, demonstrated progressive loss across all groups, with the control group exhibiting significantly accelerated softening compared to treated samples (*p* < 0.05). Notably, applications of resveratrol (RES), epigallocatechin gallate (EGCG), and tea polyphenols (TP) effectively mitigated texture degradation even under challenge with *E. coli* or *B. cinerea*. The RES-EGCG combination displayed particular synergistic efficacy, limiting hardness reduction to approximately 11% after 5 days storage, surpassing individual treatments. Similarly, the TP-chitosan integration conferred enhanced protective effects, restricting hardness loss to 33% in untreated and 45.6% in *B. cinerea*-inoculated fruit, markedly outperforming standalone applications. These preservation outcomes are attributed to the collective antimicrobial efficacy of EGCG, RES, and TP against spoilage microorganisms, thereby maintaining strawberry quality throughout storage.

As shown in Figure 4C,D,G,H, all treatment groups exhibited significantly reduced weight loss rates compared to the control group throughout the storage period (*p* < 0.05), highlighting the efficacy of these treatments in mitigating moisture loss. Notably, the combination of EGCG and RES resulted in the lowest weight loss of 14.5% by 5 days, significantly lower than the individual treatments (21.6% for EGCG and 18.7% for RES) and the control group (approximately 30%). This outcome suggests that the antioxidant properties of EGCG and the antimicrobial activity of RES work synergistically to inhibit respiration and moisture depletion. Under *E. coli* inoculation conditions, the combined treatment again demonstrated the lowest weight loss at 16.6%, underscoring its potential to suppress microbial infections that contribute to quality deterioration and moisture loss. Additionally, the TP-chitosan combination exhibited excellent moisture retention, with weight loss rates of 13% and 16% under natural storage and *B. cinerea* inoculation conditions, respectively.

In the non-inoculated groups, the spontaneous decreases in hardness and increases in weight loss of strawberries were attributed to endogenous enzymatic activity and respiration. While for strawberries infected with *B. cinerea* and *E. coli*, the decay observed during storage primarily resulted from water loss to the external environment—driven by transpiration at rot lesions and the disruption of cell membrane structure [54]. Li et al. developed a chitosan-based composite film loaded with TP nanoparticles; when applied to strawberry preservation, this film effectively mitigated the decline in strawberry hardness and the increase in weight loss [55]. Their research primarily addressed the preparation of the film and the subsequent evaluation of its preservation effect. In the present study, however, the focus shifts to the combined effect of chitosan and TP on decay caused by *B. cinerea*, a typical spoilage pathogen responsible for strawberry deterioration.

### 3.5. Epigallocatechin Gallate, Resveratrol, and Tea Polyphenols Preserved the Color of Strawberry

**Figure 5:**
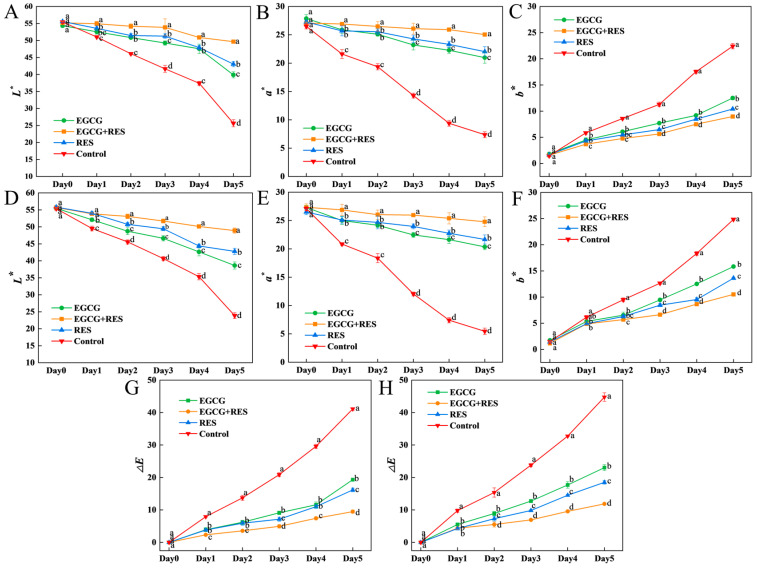
Effects of RES and EGCG on the color of strawberry infected with *E. coli*. The effects of RES and EGCG treatments on L*, a*, b*,and Δ*E* values in uninoculated (**A**–**C**,**G**) and *E. coli*-inoculated (**D**–**F**,**H**) groups. Data are presented as mean ± SD (n = 3). Different lowercase letters indicate significant differences (*p* < 0.05) between treatments on the same day. RES: Resveratrol; EGCG: Epigallocatechin Gallate.

**Figure 6:**
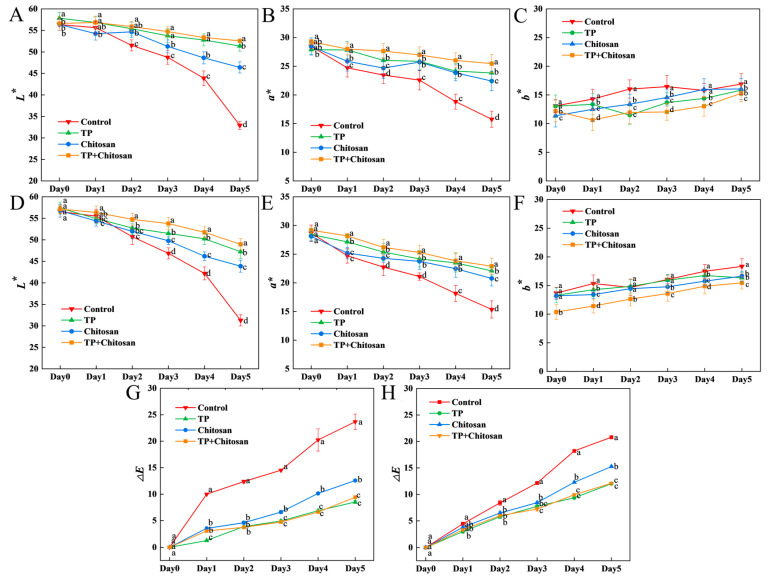
Effects of TP and chitosan on the color of strawberry infected with *B. cinerea*. The effects of TP and chitosan treatments on L*, a*, b*, and Δ*E* values in uninoculated (**A**–**C**,**G**) and *B. cinerea*-inoculated (**D**–**F**,**H**) groups. Data are presented as mean ± SD (n = 3). Different lowercase letters indicate significant differences (*p* < 0.05) between treatments on the same day. TP: Tea Polyphenols.

The color quality of strawberries typically declines during storage; however, as illustrated in Figure 5 and Figure 6, the application of EGCG, RES and TP effectively delayed this deterioration. After 5 days of storage, the L* (brightness) and a* (redness) values of strawberries in the treated groups were significantly higher than those in the control group (*p* < 0.05). Additionally, both individual and combined treatments reduced the b* (yellowness) that developed in strawberries during storage, as well as the Δ*E* (color difference) between the strawberries at the initial stage and after storage.

In both *E. coli*-inoculated and non-inoculated strawberries, the combination of EGCG and RES outperformed the individual application of either compound in mitigating the decline in L* and a* values, with the final L* and a* values increased by 10% to 15% compared to the individual treatment groups (Figure 5A,B,D,E). Compared to the control group, treatment with either EGCG or RES alleviated the accumulation of yellow color in strawberries (Figure 5C,F). During storage, among all treatments and the control group, the combination of RES and EGCG most effectively suppressed color changes in strawberries (Figure 5G,H).

The color deterioration of *E. coli*-infected strawberries can be mitigated by biocontrol agents such as the quorum-quenching enzyme AiiA, which enhances the retention of brightness and redness during storage [36]. Similarly, the phenolic compounds RES and EGCG exerted a comparable color-protective effect on *E. coli*-infected strawberries.

For strawberries either infected with *B. cinerea* or not, the combination of chitosan and TP also exhibited superior preservation of brightness (L*) and redness (a*) compared to the individual use of either component alone (Figure 6A,B,D,E). Compared to the treatment of RES and EGCG, the application of TP together with chitosan pronounced less suppression on the change in yellow color on strawberries infected with or without *B. cinerea* during the storage (Figure 6C,F). When considering the color variation between strawberries’ initial color and their color at the end of storage, TP treatment had a more significant impact than chitosan treatment (Figure 6G,H). In addition to this study, plant extracts have also been used to boost the brightness and redness of strawberries through the use of biodegradable films [56].

### 3.6. Epigallocatechin Gallate, Resveratrol, and Tea Polyphenols Elevated the Consumer Acceptance of Strawberries

**Figure 7:**
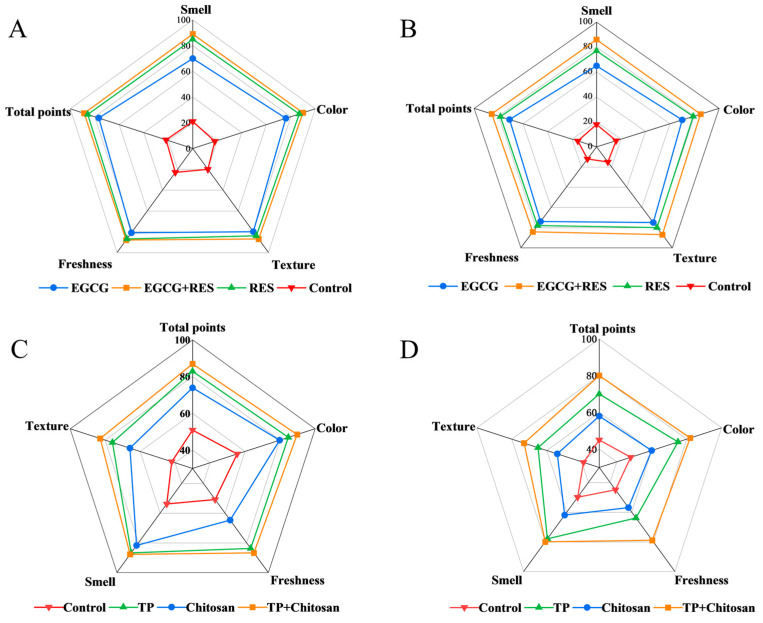
Effects of RES, EGCG, and TP on the sensory quality of strawberries. Effects of RES and EGCG in uninoculated (**A**) and *E. coli*-inoculated (**B**) strawberries. Effects of TP and chitosan in uninoculated (**C**) and *B. cinerea*-inoculated (**D**) groups. Error bars represent standard deviation (*n* = 10 panelists). RES: Resveratrol; EGCG: Epigallocatechin Gallate; TP: Tea Polyphenols.

Sensory evaluation serves as a critical measure of preservation efficacy, with higher scores indicating greater consumer acceptance [50]. The strawberries treated with EGCG or RES exhibited significantly improved sensory attributes compared to the control group. After 5 days of storage, EGCG–RES-treated strawberries outperformed the control group: they maintained better texture, minimal decay, acceptable odor and color, achieved the highest sensory scores (88.67 in natural storage, 85.56 under *E. coli* infection), and showed significantly improved consumer acceptance (Figure 7A,B).

Under natural storage conditions, the TP-chitosan combination achieved the highest sensory score of 82.8, significantly outperforming both individual treatments (75.8 for TP alone and 65.8 for chitosan alone) and the control group (Figure 7C). This advantage was particularly pronounced under *B. cinerea* infection, where the composite treatment maintained a score of 73.0, substantially higher than the individual TP and chitosan treatments, with notable improvements in strawberry hardness preservation (Figure 7D). This study investigated the consumer acceptability of phenolic compounds at their working concentrations, offering guidance for the use of these extracts in food preservation—an area previously constrained by the undesirable sensory changes these extracts induce in foods [57].

### 3.7. Epigallocatechin Gallate, Resveratrol, and Tea Polyphenols Enhanced the Flavor Quality of Strawberry

**Figure 8:**
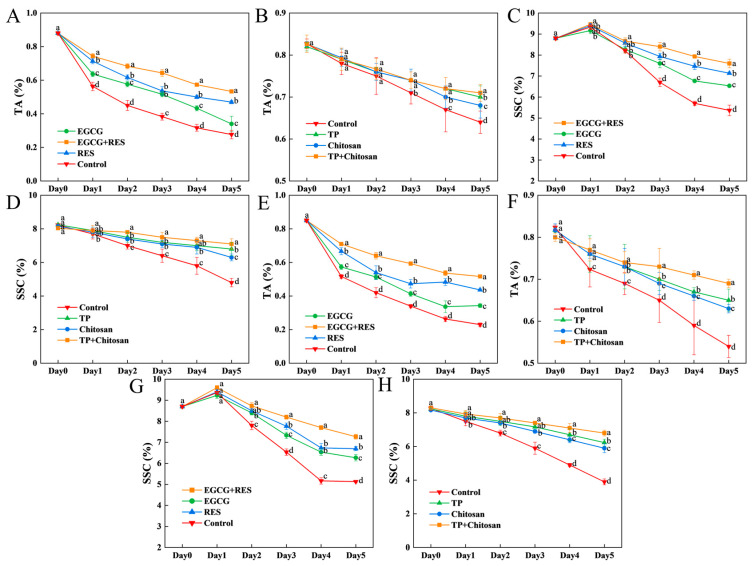
Effects of RES, EGCG, and TP on TA and SSC of strawberry. Effects of RES and EGCG on TA and SSC in uninoculated (**A**,**C**) and *E. coli*-inoculated (**E**,**G**) groups, respectively. Effects of TP and chitosan on TA and SSC in uninoculated (**B**,**D**) and *B. cinerea*-inoculated (**F**,**H**) groups, respectively. Data are presented as mean ± SD (n = 3). Different lowercase letters indicate significant differences (*p* < 0.05) between treatments within the same group. RES: Resveratrol; EGCG: Epigallocatechin Gallate; TP: Tea Polyphenols; TA: Titratable acid; SSC: soluble solids content.

The titratable acid (TA) content decreased due to the respiratory activity in strawberries during the storage. The control group exhibited the greatest reduction in TA, with content of 0.2% and 0.3% with or without infection by *E. coli* (Figure 8A,E). The EGCG–RES combination exhibited the most effective preservation, maintaining a TA content of 0.6%, significantly higher than that in the individual treatment groups and the control group. Meanwhile, the effect of TP and chitosan treatment on the TA content was less pronounced compared to the treatment with RES and EGCG. A 3-day period was required for TP or chitosan to begin acting on slowing down TA loss during strawberry storage (Figure 8B). For *B. cinerea*-infected strawberries, individual or combined use of TP and chitosan was more effective at preserving TA content than in the non-infected group (Figure 8F).

The soluble solids content (SSC), another key determinant of strawberry flavor quality, should be monitored during storage or under microbial infection [58]. Treatments with EGCG or RES effectively mitigated SSC loss, better maintaining postharvest quality compared to the control group (Figure 8C,D,G,H). The combined application of EGCG and RES was significantly more effective in preserving SSC than individual treatments, limiting total loss to only 1.0% and 1.6% in non-infected and *E.coli*-infected strawberries, respectively (Figure 8C,G). Again, the TP-chitosan combination treatment was more effective in maintaining the SSC of strawberries compared to the individual treatment groups (Figure 8D,H). Whereas for the TP and chitosan treatments, the SSC of strawberries infected with *B. cinerea* or not in control group showed little difference, indicating that TP or chitosan preserved strawberry quality not by inhibiting *B. cinerea* itself.

Previous studies developed a gallic acid-grafted N-carboxymethyl chitosan coating to improve strawberry quality by maintaining TA content, with the preservation effect of this grafted coating being more pronounced than that of N-carboxymethyl chitosan used alone [59]. As a coating for food preservation, chitosan has exhibited considerable potential. This study supplemented more data regarding the use of chitosan combined with phenolic compound in strawberry preservation; however, further research is required to develop effective chitosan-based strategies for food preservation. Notably, TP and chitosan treatment inhibited the increase in strawberry SSC during early storage, which was attributed to the enzymatic activity inherent to the strawberries [58]. Consistent with the findings of this study, Fan et al. have shown that resveratrol (RES) has the potential to retain SSC in strawberries under natural storage conditions [60]. Whereas they did not investigate the effect of RES and EGCG combination treatment on SSC in strawberries nor did they consider the microbial spoilage by *E. coli*.

## 4. Conclusions

This study investigated the anti-microbial effects of RES, EGCG, and TP and their potential role in strawberry preservation against *E. coli* and *B. cinerea*. We found that the combined use of EGCG and RES exhibited an additive effect in inhibiting the growth of *E. coli*. Furthermore, this combination significantly mitigated the deterioration of physical indices and flavor components in both naturally stored strawberries and those infected with *E. coli*. Meanwhile, TP effectively inhibited the growth and spore germination of *B. cinerea*. The combined application of TP and chitosan offered superior protection to strawberries against *B. cinerea* infection, ultimately maintaining strawberry quality at a level comparable to that of the naturally preserved controls. These findings provide experimental support for using EGCG–RES and TP–chitosan combinations as alternative strategies to preserve strawberry quality.

## Data Availability

The original contributions presented in the study are included in the article/Supplementary Materials, further inquiries can be directed to the corresponding authors.

## Supplementary Materials

The following supporting information can be downloaded at: https://www.mdpi.com/article/10.3390/foods14234142/s1, Table S1: The sensory evaluation criteria; Table S2: The mycelial diameter of three fungus treated by tea polyphenol.
