# Supplementary material for: Antimicrobial Effects of Three Plant-Derived Phenolic Compounds and Their Potential Role in Strawberry Preservation
Source: Foods. 2025 Dec 3;14(23):4142. doi: 10.3390/foods14234142 (PMC12692689; doi:10.3390/foods14234142)
Supplement: Supplementary file 1 [file foods-14-04142-s001.zip › Supplementary materials.pdf]

Supplementary date

Antimicrobial Effects of Three Plant-derived Phenolic Compounds and Their Potential Role in Strawberry Preservation

Short title: Antimicrobial Phenolics from Plants for Strawberry Preservation

Ziwei Liang[#,1], Shengshuai Li[#,1], Lanxi Zhang[1], Fengqin Wu[1], Shuyan Pu[1], Xinyue Liu[1], Yu Rao[1,*].

[#] Ziwei Liang and Shengshuai Li contributed equally to this work.

[*] Corresponding author. Emails: ryfish@163.com

[1] Food Microbiology Key Laboratory of Sichuan Province, Chengdu 610039, China

Table S1: Sensory evaluation criteria

Score	Color	Odor	Texture	Freshness
100-76	Bright red	Strong aroma	Plump and firm	Intact shape, tight skin, no damage
75-51	Slight browning	Moderate aroma	Slightly soft	Mostly intact, no decay
50-26	Moderate browning	Faint aroma	Significantly soft	Partial shrinkage/decay
25-0	Severe browning	Off-odor	Severely soft	Extensive decay

Table S2: Effects of TP on mycelial growth inhibition rates of three fungal strains

	TP concentration (g/L)	0	10.0	5.0	2.5	1.25	0.63	0.31	0.16
B. cinerea	Mycelial diameter (mm)	40	5	5	8	16	24	31	35
	Mycelial growth inhibition rate (%)	0	100	100	91.4	68.9	45.3	26.5	12.7
C. gloeosporioides	Mycelial diameter (mm)	35	5	7	13	20	26	30	32
	Mycelial growth inhibition rate (%)	0	100	93.8	72.6	47.2	28.7	15.3	8.5
A. niger	Mycelial diameter (mm)	42	5	9	17	26	32	36	38
	Mycelial growth inhibition rate (%)	0	100	88.2	65.3	39.8	23.6	12.4	5.1
